# Evaluation of the DDSolver Software Applications

**DOI:** 10.1155/2014/204925

**Published:** 2014-04-27

**Authors:** Jieyu Zuo, Yuan Gao, Nadia Bou-Chacra, Raimar Löbenberg

**Affiliations:** ^1^Faculty of Pharmacy and Pharmaceutical Sciences, University of Alberta, Edmonton, AB, Canada T6G 2E1; ^2^Department of Pharmacy, Changhai Hospital, Second Military Medical University, Shanghai 200433, China; ^3^Faculty of Pharmaceutical Sciences, University of Sao Paulo, 05508-000 Sao Paulo, SP, Brazil

## Abstract

When a new oral dosage form is developed, its dissolution behavior must be quantitatively analyzed. Dissolution analysis involves a comparison of the dissolution profiles and the application of mathematical models to describe the drug release pattern. This report aims to assess the application of the DDSolver, an Excel add-in software package, which is designed to analyze data obtained from dissolution experiments. The data used in this report were chosen from two dissolution studies. The results of the DDSolver analysis were compared with those obtained using an Excel worksheet. The comparisons among three different products obtained similarity factors (*f*_2_) of 23.21, 46.66, and 17.91 using both DDSolver and the Excel worksheet. The results differed when DDSolver and Excel were used to calculate the release exponent “*n*” in the Korsmeyer-Peppas model. Performing routine quantitative analysis proved to be much easier using the DDSolver program than an Excel spreadsheet. The use of the DDSolver program reduced the calculation time and has the potential to omit calculation errors, thus making this software package a convenient tool for dissolution comparison.

## 1. Introduction

Dissolution testing has been recognized as an important tool for both drug development and quality control because it determines the rate and extent of the drug release from orally administered pharmaceutical products. In addition, dissolution testing can also provide an* in vitro *prediction of the* in vivo* drug absorption in certain cases. Biowaivers utilize dissolution testing to assess similarity between two products, if proven to be equivalent, then bioequivalence studies are deemed to be unnecessary [1, 2]. However, when a new oral dosage form is developed, one must ensure that the drug release occurs as desired by the product specification. Mathematical models can be applied to express the dissolution data as a function of parameters related to pharmaceutical dosage to characterize the* in vitro* drug release behavior [3].

A dissolution profile is a measurement of* in vitro* drug release from a preparation in a receptacle media over a period of time. Multiple samples are normally collected at several time points. The resulting curve represents the mean cumulative drug dissolved over time. The dissolution test procedure can be differentiated into two categories: (1) if the collected sample volume is not replaced with equal amount of receptacle media, both the receptacle volume and drug are lost during sampling, and the equation for drug release quantity is *F* = (*C*_*t*_∗[*V*_or_ − (*n* − 1)*V*_*s*_])/(Dose − ∑_1_^*t*−1^ *C*_*t*_∗*V*_*s*_); (2) if the collected sample volume is replaced with equal amount of receptacle volume, only the amount of drug removed must be considered, and the equation for drug release quantity at each time point is *F* = (*C*_*t*_∗*V*_or_)/(Dose − ∑_1_^*t*−1^ *C*_*t*_∗*V*_*s*_). In these equations *F* is the quantity of drug release, *C*_*t*_ is the concentration at this time point, and *V*_or_ and *V*_*s*_ are original receptacle volume and the collected sample volume, respectively. As seen, the sample correction might be necessary and should not be omitted to achieve the actual dissolution profiles [4].

Dissolution data analysis is performed by comparing dissolution profiles statistically or using mathematical models to quantify or characterize the drug release from a pharmaceutical dose form. Most commercial statistical software programs which are used in pharmaceutical R&D are not designed for the statistical evaluation of dissolution profiles but evaluate pharmacokinetic parameters. To reduce the calculation time and to eliminate calculation errors, researchers designed the DDSolver program [5], which is a free and ready-to-use Excel plug-in program that allows the modeling of the dissolution data using 40 built-in dissolution models. In addition, this software allows a similarity analysis to be performed using well-established profile comparison approaches. The program provides an efficient data analysis report to summarize the dissolution data analysis.

This report aims to evaluate the application of the DDSolver program for analyzing dissolution data from different drug release systems. A particular focus is on the application of DDSolver to compare the dissolution profiles as described in leading regulatory guidelines [6–11]. Similarities between the different dissolution profiles were investigated according two different aspects: (1) characterization of drug release, (2)* in vitro* similarity of profiles for biowaivers applications [12]. The similarity factor (*f*_2_) [13] is a comparison method that is recommended by regulatory guidelines. As a simple measure of profile similarity, *f*_2_ is obtained by comparing the mean percentage of the drug released at each sampling time point for two curves and is defined as the logarithmic reciprocal square root transformation of 1 plus the averaged squared mean differences as follows:

(1) $$\begin{array}{c} f_{2}=50\cdot \log\left\{{\left[1+\frac{1}{n}\overset{n}{\underset{t=1}{\sum }}{\left(R_{t}-T_{t}\right)}^{2}\right]}^{-0.5}\times 100\right\}, \end{array}$$

where *n* is the number of time points and *R*_*t*_ and *T*_*t*_ are the average percentage of active pharmaceutical ingredients (API) dissolved in the reference and test products, respectively, at time *t*. The value of *f*_2_ falls between 0 and 100, and two profiles are considered to be similar when *f*_2_ ranges between 50 and 100. The FDA guideline [7] suggests that *f*_2_ could be used to compare dissolution profiles when the following criteria are satisfied: (1) sufficient sampling times are available with a minimum of 3 excluding *t* = 0; (2) the individual dosage unit for each product should be 12; (3) only a single sampling time should be considered when the percent drug release exceeds 85%; (4) the sampling times should be the same for the two products; and (5) the within-batch coefficient of variation (CV) should be below 20% for the early time points and below 10% for other time points. An *f*_2_ comparison is unnecessary when both the test and reference products exceed 85% dissolved within 15 min. Products are considered to be similar because they meet the “very rapidly dissolving” standards set by the European Medicines Agency [10]. When the within-batch CV exceeds 15%, multivariate confidence region, procedures are recommended over *f*_2_ [7, 14]. This comparison approach is built into the DDSolver program as well; however, because our study does not feature any data with a CV above 15%, this approach is not discussed in this report.

Mathematical modeling of drug release can be used in optimizing the design of novel dosage forms, elucidate the underlying drug release mechanisms, and adequately estimate the required parameters and preparation procedures for different dosage forms [15]. Many mathematical theories on dissolution behavior have been described in the literature [16–19]. The DDSolver software features 40 models built into its library that can be used to directly calculate the parameters and the appropriateness of each model. Our report investigated the drug release mechanism for gelatin nanoparticles by fitting the dissolution data with the Korsmeyer-Peppas (K-P) [20] model and its modified forms using DDSolver.

The K-P model, the so-called power law, has frequently been used to describe the drug release phenomena of various modified-release pharmaceutical dosage forms. The following equation is used for the K-P model:

(2) $$\begin{array}{c} F=kt^{n}, \end{array}$$

where *F* is the cumulative quantity of drug released at time *t*, *k* is a constant that incorporates the structural and geometric characteristics of the device, and *n* is the release exponent. The value of *n* helps to define the mechanism of drug release. The K-P model is a decision parameter to identify drug transport mechanism. The value of *n* is used to differentiate between the various drug release mechanisms [21, 22] as described in Table 1. The use of the *n* value as a criterion to discriminate dissolution mechanisms is influenced by the nature and geometries of the drug delivery system. Generally, an early portion of a release profile (*F* < 60%) is used in the model.

According to Table 1, the drug release mechanism could be separated into three cases: (1) Fickian diffusion, which could be observed in nonswelling systems or in cases where the relative relaxation time of polymer is much shorter than the characteristic diffusion time in water with water transport controlled by a concentration gradient; (2) case II transport, which refers to the erosion of the polymer chain; and (3) anomalous transport, which is intermediate to Fickian and case II transport [23].

Two modified forms of the K-P equation are used to describe the late onset in the release rate (*T*_lag_) at the beginning of the release profile [18] and the initial burst release effect (*F*_0_), respectively [24]. The equations are modified in the following manner:

(3) $$\begin{array}{c} F=k{\left(t-T_{\text{lag}}\right)}^{n},\\ F=kt^{n}+F_{0}. \end{array}$$

Several issues should be considered when fitting the dissolution data to a suitable model. The theoretical calculations should be compared with the experimental results to perform a simulation adjustment of the model parameters. It is possible to obtain a good fit even if the model is inappropriate for the drug delivery system. In addition, if adequate experimental evidence is provided, one should use that evidence no matter how well theoretical models agree with the dissolution results [15]. The selection of suitable criteria for fitting a model from a number of statistical criteria that are featured in the DDSolver program is critical. The three most important criteria for the selection of a dissolution model include the following: the adjusted coefficient of determination (*R*_adjusted_^2^) [3], the Akaike Information Criterion (AIC) [25], and the model selection criterion (MSC) [26]. These criteria produce different values for assessing the appropriateness of a model.

In addition, several other useful and efficient modules are built into the DDSolver program, including a dissolution sampling-volume correction and a calibration curve analysis. This report focuses on the analysis of dissolution data to evaluate the application of the DDSolver program to two dissolution studies: (1) the comparison of dissolution profiles between different products, (2) fitting of drug release data to the K-P model and its modifications.

## 2. Materials and Methods 

The model data used in this paper were obtained from two recent research projects of the authors.

**Figure 1:**
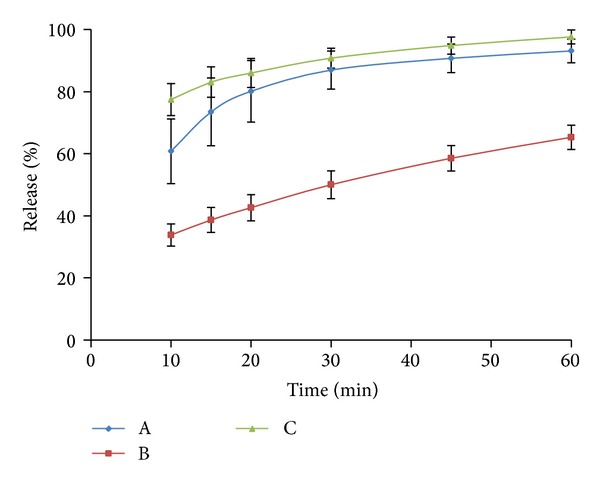
Dissolution behavior of three 250 mg amoxicillin products in simulated intestinal fluid.

**Figure 2:**
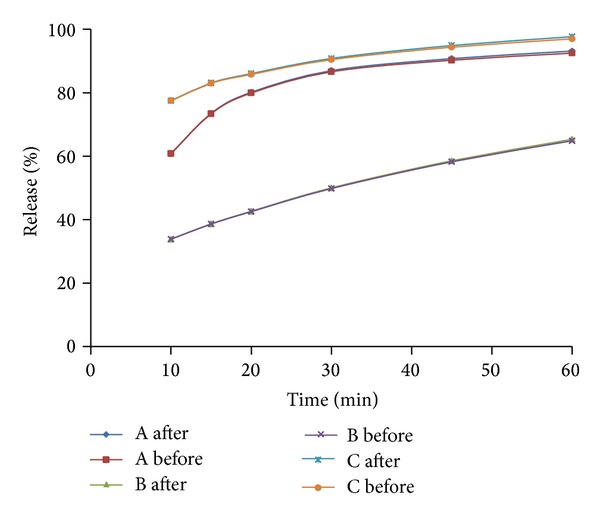
Dissolution profiles of three 250 mg amoxicillin products in simulated intestinal fluid before or after sample correction.

The data used to evaluate the dissolution profiles were obtained from the dissolution study of three different 250 mg amoxicillin capsules purchased from China, which are labeled A, B, and C. The test units were placed in a VK 7020 dissolution tester with six vessels, a VK 8000 autosampler station (Agilent Technologies, Carey, NC), and a USP apparatus 2. The Japanese Pharmacopeia Basket Sinkers (Quality Lab Accessories, Brigewater, NJ) which are compliant with USP were utilized. In the dissolution process, 1.25 mL sample was collected at 10, 15, 20, 30, 45, and 60 min without sample replacement from a 900 mL receptacle volume, respectively. The profiles after sample correction are shown in Figure 1 and used for profile comparison.  Figure 2 shows profiles in the presences and absences of sample correction.

**Figure 3:**
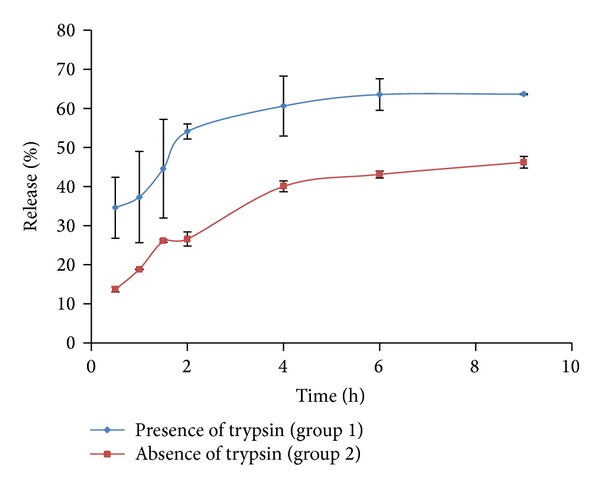
Dissolution profile of gelatin nanoparticles in the presence or absence of trypsin.

Two groups of gelatin nanoparticle data were used to determine which K-P model provides the best fit. The detailed materials and methods of this study were published by Gao, et al. [27]. In brief, the drug release from gelatin nanoparticles was determined in the presence of trypsin named as group 1, whereas in the second case, drug release from gelatin nanoparticles was measured in the absence of trypsin regarded as group 2, as shown Figure 3. All of the other conditions were held constant; it was expected to see different drug release from these nanoparticles because trypsin will cause a forced degradation of the nanoparticles. The cutoff values of the exponent *n* used to distinguish between the different drug release mechanisms were *n* = 0.43 and *n* = 0.85. An Excel (Microsoft Corporation, Redmond, WA, USA) worksheet was used as a control computing method for comparison with the results obtained using the DDSolver program. All data were entered into Excel according to the example format for each built-in module of the DDSolver. The relevant parameters were calculated following the equations step-by-step utilizing an Excel spreadsheet. The *R*_adjusted_^2^ value was used as the model selection criterion with the best model exhibiting the *R*_adjusted_^2^ value closest to 1.

## 3. Results and Discussion

### 3.1. Sample Correction

Figure 2 shows that slight differences will be observed in the presence or absence of sample correction which is expected. In general, the amount of sample volume removed without replacement impacts the dissolution result. The sample correction can be applied mathematically either to the raw concentration (obtained from sample analysis) or as percent released, as performed in this study.

### 3.2. Profile Comparison

The profiles in Figure 1 were compared with *f*_2_ statistic in which only one drug release measurement of the tested product is allowed to exceed 85%. Therefore, the time points chosen for tested products, A and B, A and C, and B and C groups, were 10 to 30 min, 10 to 20 min, and 10 to 20 min, respectively. The values of *f*_2_ were 23.21, 46.66, and 17.91 for these groups, respectively, which suggests that the three products were dissimilar. However, if all of the data points collected during the dissolution process were considered, values of 24.08, 52.81, and 19.67 were observed, which differs from those obtained using only one measurement over 85%. A comparison of the products A and C reveals that the dissolution results were similar. The same results were obtained with Excel and DDSolver.

When using Excel to calculate the *f*_2_ value, the number of time points *n* used in the equation must be carefully accounted for, because *n* can vary among comparison groups and may not equal the number of time points collected during the experiment. Excel calculates the *f*_2_ value step-by-step using the equation and can thus only compare two products at once. Accordingly, with several different products, the calculations between each product may require a significant amount of time. Hence, when generating a profile comparison, the DDSolver program could reduce the errors and calculation time that are necessary with Excel.

*f*_2_ is actually insensitive to the shape of the dissolution profiles and is difficult to assess both type I and type II errors because there is no mathematical formula included for the statistical distribution of *f*_2_ [5, 28], which is the major drawback of *f*_2_ [29].

The bootstrap method is proposed as a tool to estimate the statistical distribution of the data and employ a confidence interval approach of *f*_2_ [28]. Bootstrap of *f*_2_ generates a new population of dissolution profiles through random samples with replacement from 12 units of the test and reference batches, respectively [28, 29]. It is possible to assess the similarity of dissolution profiles with large variability if the data populations are identically distributed. Compared to *f*_2_, bootstrap-based *f*_2_ is more accurate in similarity comparison of dissolution profiles and especially important if the *f*_2_ value is less than 60 [30]. Building the bootstrap based *f*_2_ module into DDSolver makes the similarity comparison of dissolution profiles convenient and accurate.

### 3.3. Model Fitting

Most of* in vitro* kinetics of drugs released from nanoparticles under various conditions can be described by the K-P model.

The K-P and the modified K-P models were investigated to identify the most appropriate model to describe the* in vitro* drug release kinetics for the selected nanoparticle formulations. The tested drug delivery system was gelatin-nanoparticles in which gelatin was used as a hydrophilic carrier, which is bioerodible and can swell.

From the results, *n* values outputted by K-P, K-P *T*_lag_, and K-P  *F*_0_ models for group 1 (gelatin nanoparticles in the presence of trypsin) were 0.343, 2.942, and 2.291 with *R*_adjusted_^2^ were 0.812, 0.899, and 0.995, respectively. For group 2 (gelatin nanoparticles in the absence of trypsin), the K-P, K-P   *T*_lag_, and K-P  *F*_0_ models that outputted the *n* as 0.392, 0.322, and 0.055 with *R*_adjusted_^2^ were 0.949, 0.958, and 0.968, respectively. The *n* value used to discriminate between diffusion, anomalous transport, and erosion was 0.43 and 0.85, respectively. According to the results, the K-P  *F*_0_ model and drug release by erosion were appropriate for group 1, whereas the K-P   *T*_lag_ model should be used for group 2. This is because *F*_0_ value of the model for group 2 is negative (*F*_0_ = −199.0), even if *R*_adjusted_^2^ is the most close to 1.

For group 1, with the presence of trypsin, the enzymatic degradation of nanoparticles should be taken into consideration. Generally, two erosion mechanisms exist: heterogeneous and homogeneous erosion of a polymer matrix. The heterogeneous erosion is surface erosion with degradation, which only happens at the surface of a polymer matrix. This erosion most likely takes place in hydrophobic polymers, as water is excluded. As hydrophilic polymers absorb water, they favor homogeneous erosion (bulk erosion) which is the result of degradation occurring through the polymer matrix [31, 32]. As gelatin is a kind of hydrophilic polymers, the degradation of gelatin nanoparticles induced by trypsin could be defined as homogeneous erosion, and the dissolution behavior of group 1 can be characterized as having an initial burst release [15]. Thus, the K-P *F*_0_ model exhibited drug release by erosion mechanism which is a more appropriate model for group 1.

For group 2, in the absence of trypsin, polymer swelling may play an important role in the control of drug release from the gelatin nanoparticles. Then polymer swelling should also be taken into consideration because it may increase the length of the diffusion pathways, decrease the drug concentration gradients, and potentially decrease the drug release rate [15]. Therefore, the K-P *T*_lag_ model was found to be an appropriate model to describe the drug release from gelatin nanoparticles form group 2 as well as the statistical result obtained by DDSolver.

When Excel was used to obtain the *n* value for the K-P model, the experimental data were plotted on a logarithmic scale according to the equation, and the *n* value was determined using the slope [33]. The DDSolver program uses the nonlinear least-squares curve-fitting technique to estimate such parameters by fitting the nontransformed dissolution data to the model. DDSolver employs the Nelder-Mead simplex algorithm, which is considered to be the most robust minimization algorithm [34], to minimize the objective functions, sum of squares or weighted sum of squares, and then obtain the best parameters.

The results for the two groups obtained by Excel using the K-P model were 0.309 and 0.794 and 0.437 and 0.963 for the *n* and *R*_adjusted_^2^ values, respectively. The results obtained using Excel differed slightly from those obtained using the DDSolver program. In particular, the *n* values in the K-P model estimated by Excel and DDSolver for group 2 were 0.437 and 0.392, respectively. Because the *n* value used to assess the drug release mechanism of the nanoparticles was 0.43, the difference in the *n* values indicated that the drug release mechanism of group 2 was either anomalous transport (according to Excel) or the Fickian diffusion mechanism (according to the DDSolver). As explained earlier, the different results from two methods are because when using Excel, the nonlinear data were first transformed to create a linear relationship and then were analyzed with linear regression while DDSolver used nonlinear optimization methods to facilitate the modeling of dissolution data [5]. For nonlinear data, using nonlinear regression is more sensitive than transforming the data to create a linear relationship because the transformation may distort experimental errors [3].

In addition, the use of Excel to estimate the modified K-P model parameters proved to be difficult. Future work has to focus on additional calculation approaches that can minimize the errors in the model parameter values while describing the dissolution behavior.

### 3.4. Model Selection

Several other mathematical dissolution models are built into the DDSolver program to estimate the drug release mechanisms, such as the Hixson-Crowell (H-C) model [34, 35], Baker-Lonsdale (B-L) model [36, 37], and Hopfenberg model [38]. Because the degradation of gelatin nanoparticles is characterized by a homogeneous erosion as mentioned above, the H-C model, which describes the release profile of drug particles with a diminishing surface area during dissolution [39], and the Hopfenberg model, which describes the heterogeneous erosion [40], cannot be applied. Thus, this report only discusses the B-L model.

The B-L model was developed from the Higuchi Model [41, 42] to describe drug release as a diffusion process based on the Fickian law. It can be represented by the following expression:

(4) $$\begin{array}{c} \frac{3}{2}\left[1-{\left(1-\frac{F}{100}\right)}^{2/3}\right]-\frac{F}{100}=\frac{3DC_{s}}{r_{0}^{2}C_{0}}t, \end{array}$$

where *F* is the cumulative quantity of drug released at time *t*, *D* is the diffusion coefficient, *C*_*s*_ is the saturation solubility, *r*_0_ is the initial radius of a sphere, and *C*_0_ is the initial drug loading in the matrix. DDSolver, uses *k*_BL_ rather than 3*DC*_*s*_/*r*_0_^2^ *C*_0_ in the equation. As these parameters are all constants, when using DDSolver to fit the data to the B-L model, it is not necessary to consider and input the value of these parameters and the output only provides the value of *k*_BL_.

The *R*_adjusted_^2^ values obtained for group 1 (gelatin nanoparticles in the presence of trypsin) using the B-L model and B-L model were 0.376 and 0.798, respectively, and the values were 0.945 and 0.940 for group 2 as determined by DDSolver. The results showed that the B-L model did not provide a good fit for group 1, which means that it did not follow Fickian diffusion. In contrast, *R*_adjusted_^2^ value of the B-L model for group 2 (gelatin nanoparticles in the absence of trypsin) was nearly 1, which illustrated that this group may follow Fickian diffusion. Both of these findings were consistent with the kinetics of drug release from gelatin nanoparticles defined by the K-P model using case II transport and diffusion mechanisms, respectively.

### 3.5. Report Features in DDSolver

**Figure 4:**
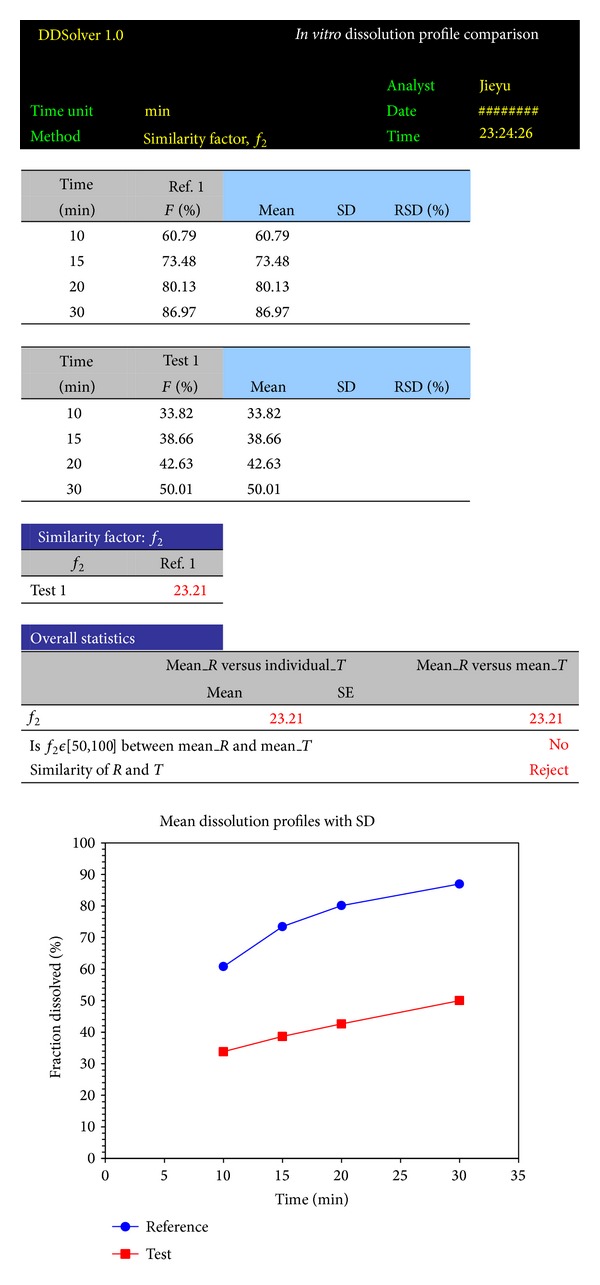
Example DDSolver report of profile comparison.

**Figure 5:**
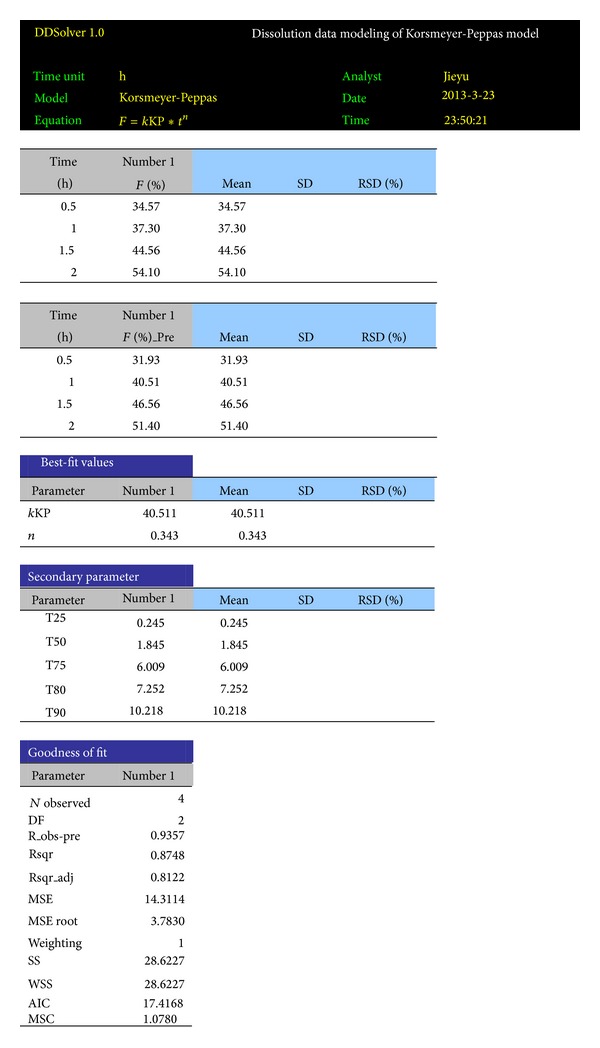
Example DDSolver report of model selection.

Outputs of DDSolver are displayed for each analysis as text and graphic mode in Microsoft Excel dataset or as text only in Word which is very convenient for the user because it can be incorporated in customized reports. The copies of DDSolver reports of a spreadsheet for profile comparison and model selection are shown in Figures 4 and 5, respectively.

## 4. Conclusion

DDSolver is a free calculation and/or statistic program used to analyze dissolution data or fit drug release data. Because DDSolver can aid in reducing calculation errors and calculation time, it is a suitable tool for day-to-day comparisons of dissolution data.

Researchers must understand each part of the program and the various models prior to using the program from both statistical and pharmaceutical aspects.

## Figures and Tables

**Table 1:** Exponent *n* of the power law and drug release mechanism from polymeric controlled delivery systems of different geometries.

Exponent, *n*			Drug release mechanism
Thin film	Cylinder	Sphere	
0.5	0.45	0.43	Fickian diffusion
0.5 < *n*< 1.0	0.45 < *n*< 0.89	0.43 < *n*< 0.85	Anomalous transport
1.0	0.89	0.85	Case II transport
